# The proneural subtype is not associated with survival benefit from bevacizumab in newly diagnosed glioblastoma: a secondary analysis of the GLARIUS trial

**DOI:** 10.1007/s11060-023-04470-9

**Published:** 2023-10-03

**Authors:** Johannes Weller, Thomas Zeyen, Niklas Schäfer, Christina Schaub, Anna-Laura Potthoff, Joachim P. Steinbach, Peter Hau, Clemens Seidel, Roland Goldbrunner, Ghazaleh Tabatabai, Hartmut Vatter, Theophilos Tzaridis, Matthias Schneider, Ulrich Herrlinger

**Affiliations:** 1 Division of Clinical Neurooncology, Department of Neurology, University Hospital Bonn, Bonn, Germany; 2 Department of Neurosurgery, University Hospital Bonn, Bonn, Germany; 3 Dr. Senckenberg Institute of Neurooncology, German Cancer Consortium (DKTK), partner site Frankfurt, University of Frankfurt, Frankfurt, Germany; 4 Department of Neurology and Wilhelm Sander NeuroOncology Unit, University Hospital Regensburg, Regensburg, Germany; 5 Department of Radiation Oncology, University of Leipzig Medical Center, Leipzig, Germany; 6 Department of Neurosurgery, University of Cologne, Cologne, Germany; 7 Department of Neurology and Interdisciplinary Neurooncology, Hertie Institute for Clinical Brain Research, Center for Neuro-Oncology, Comprehensive Cancer Center Tübingen-Stuttgart, partner site Tübingen, University Hospital Tübingen, German Cancer Consortium (DKTK), Eberhard Karls University, Tübingen, Germany

**Keywords:** Glioblastoma, Newly diagnosed glioblastoma, Bevacizumab, Gene expression

## Abstract

**Purpose:**

The AVAglio trial reported a significant survival benefit for first line bevacizumab treatment in patients with IDH wildtype glioblastoma of the proneural gene expression subtype. We here aim to replicate these findings in an independent trial cohort.

**Methods:**

We evaluate the treatment benefit of bevacizumab according to gene expression subtypes of pretreatment tumor samples (n = 123) in the GLARIUS trial (NCT00967330) for MGMT unmethylated glioblastoma patients with Kaplan-Meier analyses, log-rank tests and Cox regression models.

**Results:**

Employing the Phillips classifier, bevacizumab conferred a significant PFS advantage in patients with proneural IDH wild-type tumors (10.4 vs. 6.0 months, p = 0.002), but no OS advantage (16.4 vs. 17.4 months, p = 0.6). Multivariable analysis adjusting for prognostic covariates confirmed the absence of a significant OS advantage from bevacizumab (hazard ratio, 1.05, 95% CI, 0.42 to 2.64; p = 0.14). Further, there was no interaction between the proneural subtype and treatment arm (p = 0.15). These results were confirmed in analyses of tumor subgroups according to the Verhaak classifier.

**Conclusion:**

In contrast to AVAglio, glioblastoma gene expression subgroups were not associated with a differential OS benefit from first-line bevacizumab in the GLARIUS trial.

## Introduction

Glioblastoma is the most common and aggressive adult primary brain tumor [1]. Glioblastomas display microvascular proliferation and express elevated vascular endothelial growth factor (VEGF), which plays a key role in tumor neovascularization and growth [2]. The humanized anti-VEGF monoclonal antibody bevacizumab is approved for recurrent glioblastoma treatment in many countries based on response rate and prolongation of progression-free survival (PFS). The addition of bevacizumab to standard-of-care treatment in two randomized first-line phase III trials – AVAglio and RTOG-0825 – reported a longer median PFS, while no overall survival (OS) benefit was observed [3, 4]. A retrospective analysis of AVAglio investigated bevacizumab efficacy in gene expression subgroups and reported a significant OS and PFS advantage for patients with proneural tumors according to the Phillips and the Verhaak classification [5, 6] based on transcriptional patterns. Patients with mesenchymal tumors derived only a PFS, but no OS benefit, and patients with tumors belonging to the proliferative subclass did not derive any survival benefit from bevacizumab therapy [7]. Although eagerly awaited, no validation of these findings has been published yet [8–10].

Here, we aim to replicate these findings and analyze the potential impact of glioblastoma gene expression subgroups on the benefit from first-line treatment with bevacizumab in an independent study cohort of *MGMT*-unmethylated glioblastoma [11].

## Methods

The GLARIUS trial, a randomized phase II trial (ClinicalTrials.gov NCT00967330), recruited 170 patients (modified intention-to-treat population used for analysis of the primary endpoint) aged 18 or older with newly diagnosed glioblastoma harboring an unmethylated MGMT promotor and with a Karnofsky performance status (KPS) of 70% or higher [11]. Patients were recruited between June 2010 and August 2012 and randomized to standard temozolomide concomitant to radiotherapy followed by six courses of temozolomide, or standard radiotherapy with concomitant bevacizumab every 2 weeks followed by bevacizumab and irinotecan every 2 weeks.

Gene expression subgroups of the GLARIUS biomarker cohort with IDH wildtype glioblastoma have been published and were accessed through the Gene Expression Omnibus (GEO) database, access number GSE150615 [12]. These data were derived from baseline formalin-fixed, paraffin-embedded samples of isocitrate dehydrogenase-1 wildtype tumors that were collected and RNA was extracted and run on a customized glioblastoma panel comprising 814 features on the NanoString gene expression platform. No further selection criteria were applied apart from tissue availability. After correction, preprocessing, sample-wise normalization and conversion to z-scores, Phillips and Verhaak subtypes had been assigned [5, 6, 12].

### Statistical analysis

Baseline characteristics of the biomarker and trial cohorts were compared with Fisher’s exact test, chi-square test, and Mann-Whitney U test, where appropriate. Outcome analyses used Kaplan-Meier plots, log-rank tests and Cox proportional hazard models. Multivariable Cox proportional hazard models included the following covariates: age (years), sex, corticosteroid use at baseline (yes/no), extent of resection (biopsy, partial resection or complete resection of contrast-enhancing tumor volume in T1 MRI sequence), Karnofsky performance status (70–80 vs. 90–100), and Mini-Mental State Examination score (< 27 vs. ≥ 27) [7]. Due to the confirmatory nature of this analysis and to decrease the probability of type 2 errors, statistical significance was defined as p < 0.1 and all analyses were two-sided. Statistical analyses were carried out with R (version 4.2.1, The R Foundation for Statistical Computing, https://www.r-project.org, packages survminer and survival).

## Results

**Fig. 1:**
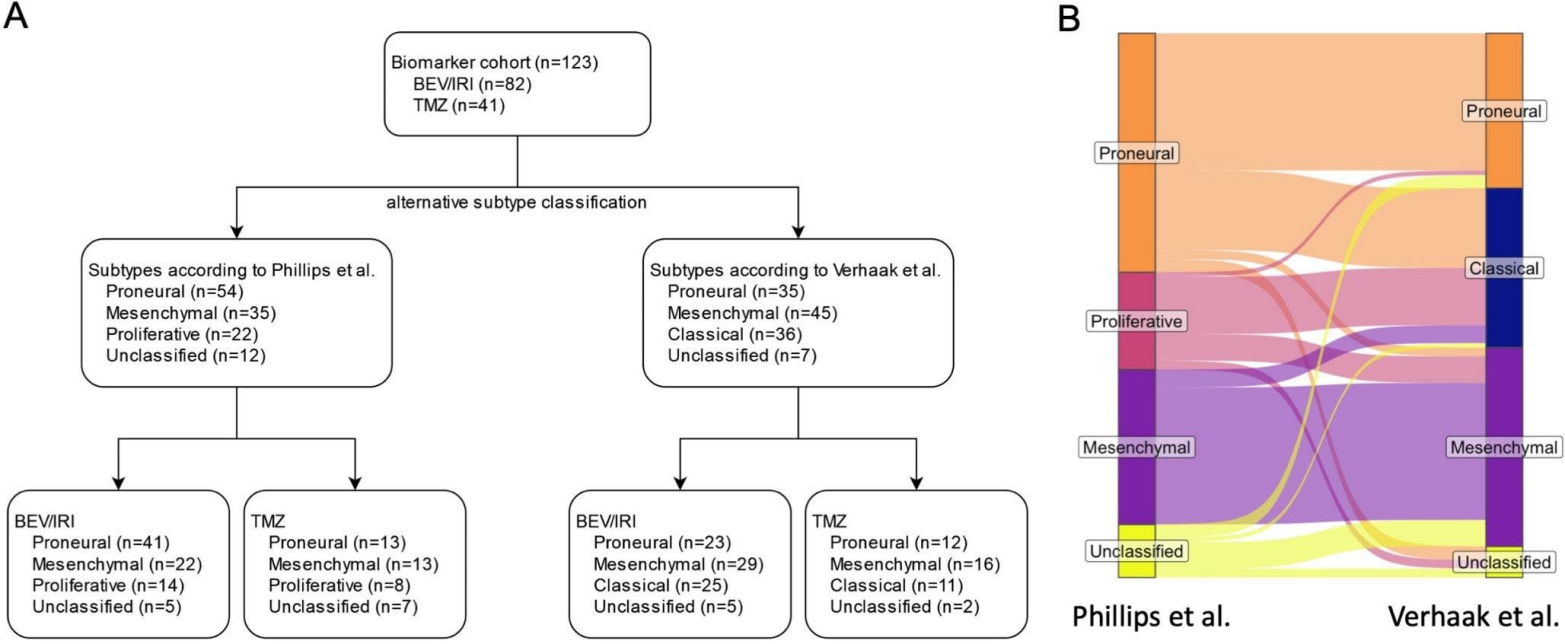
Patient flow and gene expression subgroups. A: patient flow of included patients with newly diagnosed IDH wildtype glioblastoma. B: concordance of gene expression subgroups between classifications according to the Phillips and Verhaak classifiers. Abbreviations: BEV, bevacizumab; IRI, irinotecan; TMZ, temozolomide

The biomarker cohort of IDH wildtype glioblastoma patients consists of 123 of the 170 patients treated in the GLARIUS trial (bevacizumab/irinotecan arm, n = 82; temozolomide arm, n = 41). Baseline demographics and clinical characteristics of the biomarker cohort are shown in Table 1 and were similar to the entire GLARIUS cohort. Tumors were classified as proneural in 43.9% (n = 54), mesenchymal in 28.5% (n = 35) and proliferative in 17.9% (n = 22) according to the Phillips classification [5], while 9.8% (n = 12) were unclassified. Employing the Verhaak classification [6], the proneural subtype was present in 28.5% (n = 35), while mesenchymal, classical and unclassified subtypes were found in 36.6% (n = 45), 29.3% (n = 36) and 5.7% (n = 7) of cases, respectively (Fig. 1). There was high concordance between classifications for the proneural and mesenchymal subtypes, while the Verhaak classical subtype contained the majority of proliferative samples but also proneural samples (Fig. 1B).

**Table 1:** Baseline patient demographics and clinical characteristics of the biomarker cohort and the entire trial cohort

Characteristics	Biomarker cohort (n = 123)	GLARIUS (n = 170)	p
Median age, years (IQR)	56 (49–63)	56 (39–63)	0.60
Female sex, n (%)	41 (32.9%)	56 (33.3%)	1.0
Glucocorticoids at baseline, n (%)	22 (17.9%)	32 (18.8%)	0.96
Median KPS (IQR)	90 (90–100)	90 (90–100)	0.48
Extent of resection, n (%):			0.93
Complete resection	61 (50%)	82 (48.5%)	
Partial resection	60 (49.2%)	85 (50.3%)	
Open biopsy	1 (0.8%)	2 (1.2%)	
MMSE ≥ 27, n (%)	102 (85%)	138 (83.1%)	0.79

Abbreviations: IQR, interquartile range; KPS, Karnofsky Performance Status; MMSE, Mini-Mental State Examination.

**Fig. 2:**
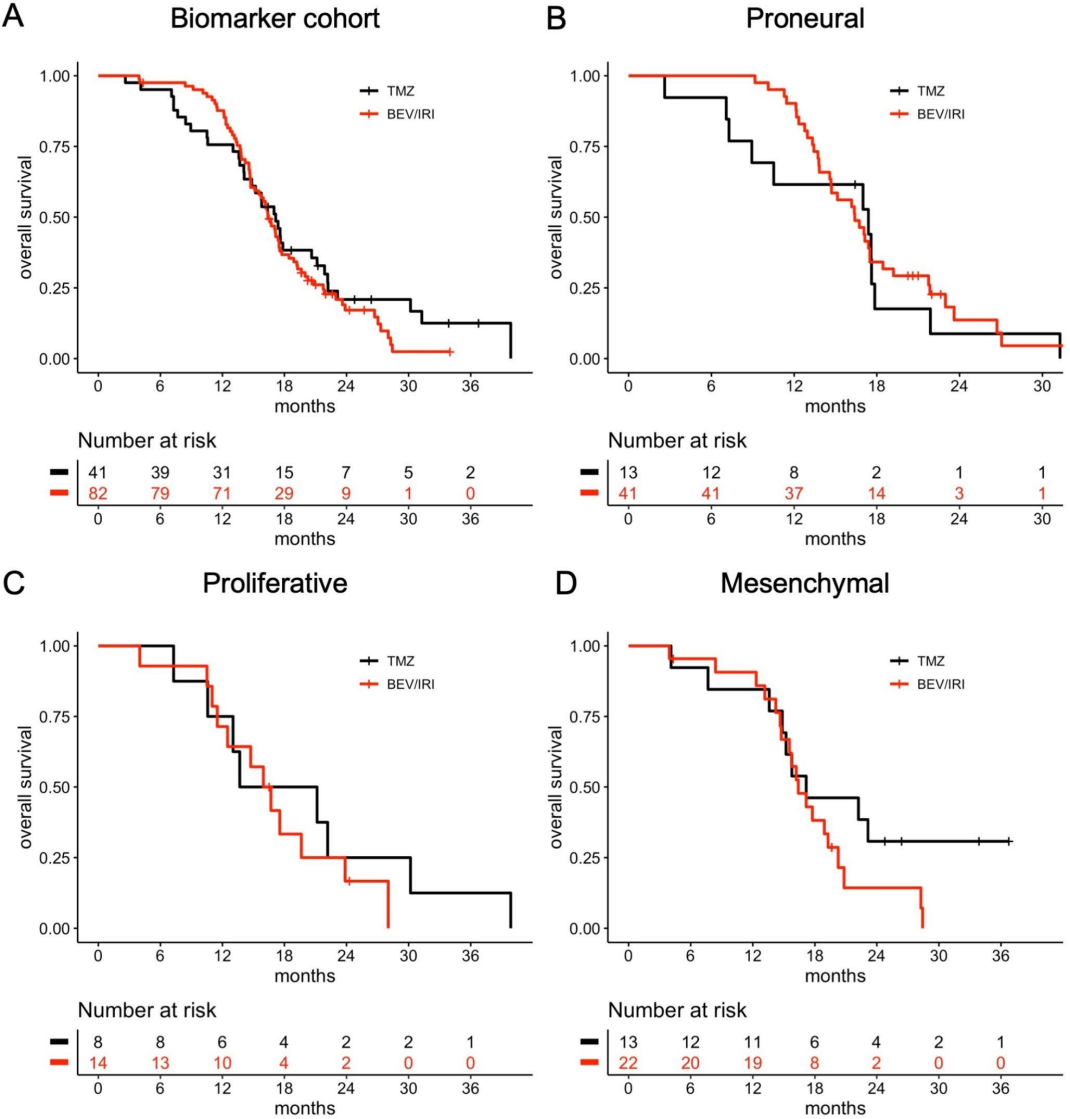
Overall survival of the biomarker cohort and glioblastoma subgroups according to treatment arm. A: biomarker cohort. B-D: glioblastoma subgroups according to the Phillips classifier. B: proneural subtype, C: proliferative subtype, D: mesenchymal subtype. Abbreviations: BEV, bevacizumab; IRI, irinotecan; TMZ, temozolomide

**Fig. 3:**
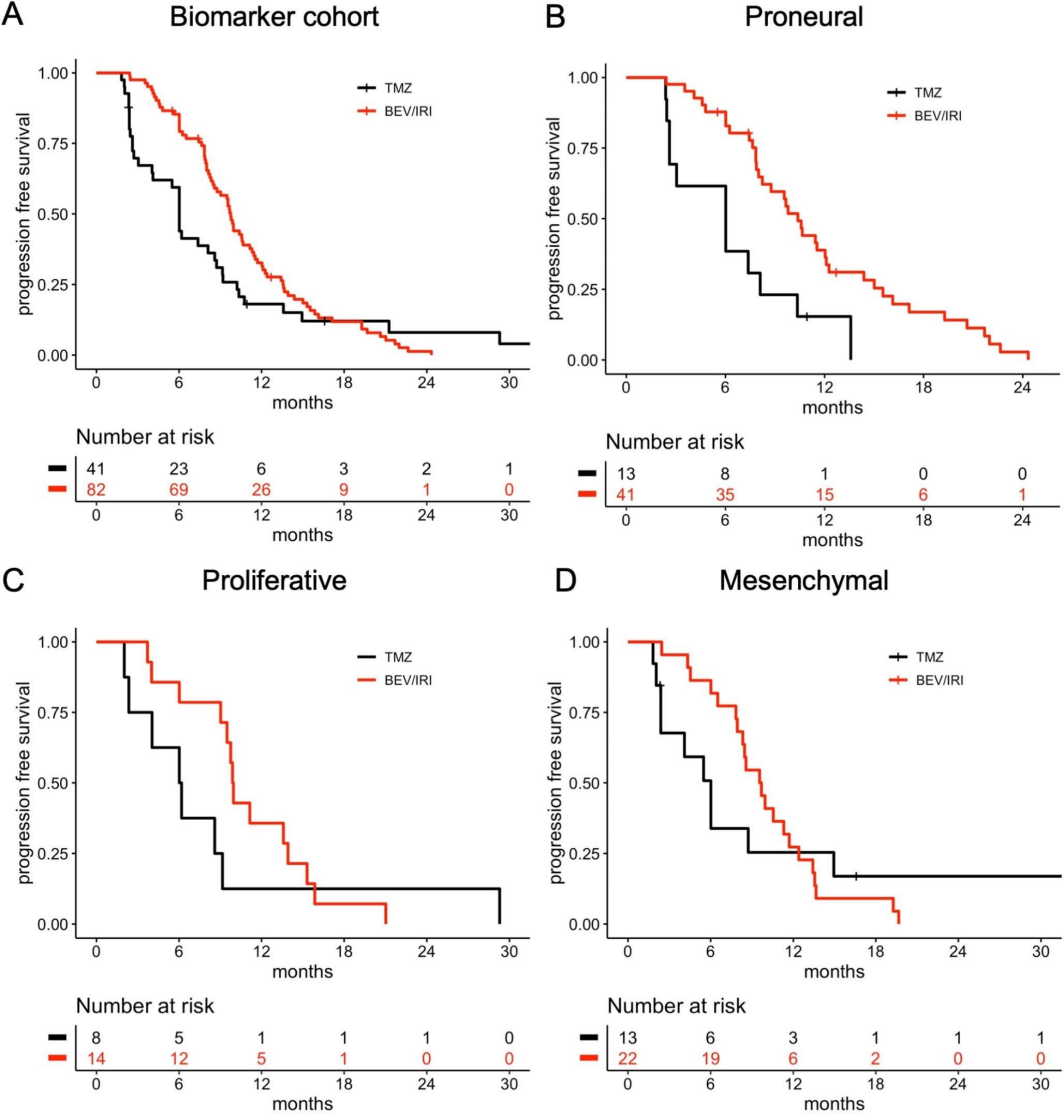
Progression-free survival of the biomarker cohort and glioblastoma subgroups according to treatment arm. A: biomarker cohort. B-D: glioblastoma subgroups according to the Phillips classifier. B: proneural subtype, C: proliferative subtype, D: mesenchymal subtype. Abbreviations: BEV, bevacizumab; IRI, irinotecan; TMZ, temozolomide

To validate the previously reported subtype-specific survival benefit of bevacizumab, outcome analyses were performed stratified for the Phillips classification [7]. Kaplan Meier plots for the biomarker cohort and the different Phillips subtypes depicting OS and PFS according to treatment arm are shown in Figs. 2 and 3. Compared to the standard arm, there was no increase in OS for proneural (median 16.4 vs. 17.4 months, p = 0.6, logrank test), proliferative (median 16.3 vs. 17.4 months; p = 0.5) and mesenchymal subtypes (median 16.4 vs. 17.2 months, p = 0.2; Fig. 2). These results were confirmed in univariable Cox regression analyses (all p > 0.1). The proneural subtype was associated with longer PFS in the bevacizumab arm (median 10.4 vs. 6.0 months; p = 0.002, log-rank test) and not among proliferative (median 9.9 vs. 6.1 months, p = 0.3) and mesenchymal tumors (median 9.6 vs. 6.0 months; p = 0.8), but these subgroups were smaller and visual inspection of the Kaplan Meier plots suggested a potentially similar PFS difference (Fig. 3).

For multivariable analysis, patients were divided into proneural and non-proneural subtypes and known prognostic factors were incorporated as reported by Sandmann et al. [7]. In both the proneural and non-proneural subtypes, the administration of bevacizumab was not associated with extended OS (proneural: adusted hazard ratio [aHR] 1.05, 95% CI 0.42–2.64, p = 0.84; non-proneural: aHR 1.73, 95% CI 0.84–3.59, p = 0.14). Formal testing of the interaction between proneural subtype and treatment arm confirmed the absence of a differential OS benefit from bevacizumab (p = 0.15).

**Fig. 4:**
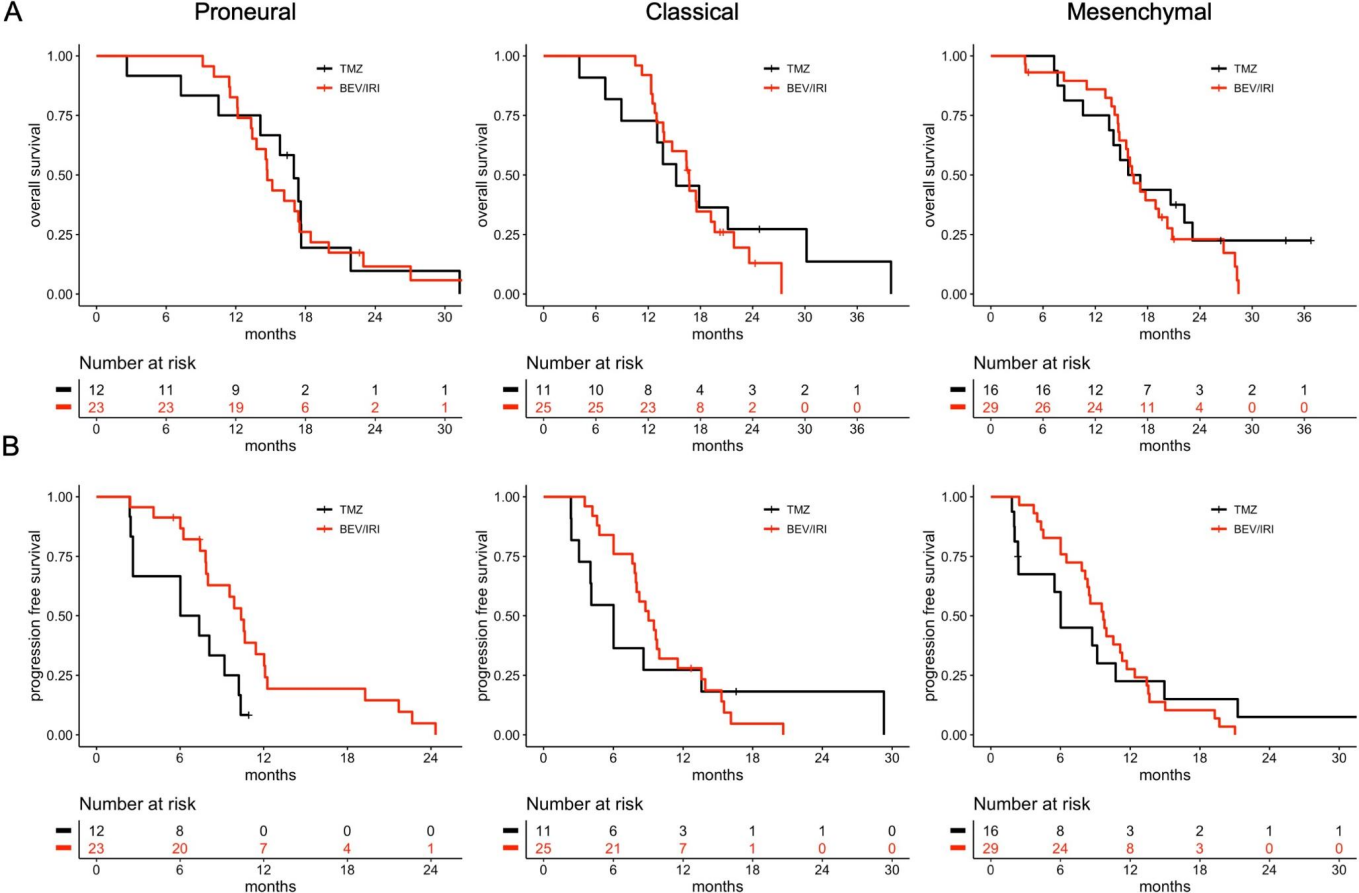
Overall survival and progression-free survival of glioblastoma subgroups according to the Verhaak classifier and treatment arm. A: Overall survival of proneural, classical and mesenchymal tumors according to the Verhaak classifier. B: Progression-free survival of proneural, classical and mesenchymal tumors. Abbreviations: BEV, bevacizumab; IRI, irinotecan; TMZ, temozolomide

Additional analyses employing the Verhaak classification confirmed the absence of a significant OS benefit for bevacizumab in the proneural, classical and mesenchymal subtypes (log-rank test and univariable Cox regression analysis, all p > 0.1; Fig. 4A). Multivariable Cox regression analysis adjusted for the same covariables found no OS benefit from bevacizumab for the proneural (aHR 1.52, 95% CI 0.50–4.58, p = 0.46) and non-proneural subtypes (aHR 1.56, 95% CI 0.82–2.97, p = 0.17). Again, no interaction between the proneural subtype and treatment arm was found for OS (p = 0.68). A significant PFS benefit from bevacizumab was again present in the proneural subgroup (10.4 vs. 6.7 months, p = 0.01) and not in classical (9.0 vs. 6.0 months, p = 0.8) and mesenchymal tumors (9.7 vs. 6.0 months, p = 0.9), but these subgroups were smaller and visual inspection of the Kaplan Meier plots suggested a PFS difference in the latter (Fig. 4B).

## Discussion

In the GLARIUS trial, none of the IDH wildtype glioblastoma subgroups defined by gene expression analysis had a differential OS benefit from first-line bevacizumab treatment. Thus, previous results reporting an OS benefit from bevacizumab for the proneural subgroup could not be confirmed [7]. This observation is in line with single-cell data, challenging the concept of glioblastoma subtypes with the observation that within a single tumor, glioblastoma cells exist in different cellular states with considerable plasticity [13]. Regarding a potentially different PFS benefit, we were able to confirm the prolonged PFS for proneural tumors, while there was no statistically significant difference in the other subgroups. However, due to the smaller subgroup sizes and the graphical PFS difference in Kaplan Meier plots, the absence of evidence for a significant PFS benefit in proliferative and mesenchymal tumors should not be taken for evidence of its absence.

Extensive angiogenesis is a typical feature of glioblastoma, and antiangiogenic therapy has been the most investigated strategy for glioblastoma in the last decade. Overexpression of tyrosine kinase receptors such as VEGF receptors is involved in glioma angiogenesis. While the monoclonal VEGF-antibody bevacizumab was approved for recurrent glioblastoma in some countries based on prolonged PFS and clinical benefit, such as the reduction of steroid need and neurological symptoms, it did not prolong OS in several phase 3 clinical trials - both in newly diagnosed and recurrent disease [3, 4, 11, 14]. Following these disappointing results, different mechanisms of resistance to antiangiogenic therapy have been identified such as compensatory angiogenic signaling or vessel co-option [15]. Some initiatives set out to identify subgroups potentially deriving an OS benefit from bevacizumab, such as the report from Sandmann and colleagues associating the proneural subtype with increased OS from bevacizumab, which seems counterintuitive as the mesenchymal and not the proneural subtype shows elevated angiogenic markers including VEGF [7]. However, mesenchymal gene enrichment was associated with shorter OS in the bevacizumab arm of RTOG 0825, supporting the observed lower sensitivity of mesenchymal tumors to bevacizumab [4]. Sulman and colleagues planned to evaluate the impact of a mesenchymal gene signature on bevacizumab treatment response, but their results remain yet to be reported [16]. Another study containing data from the GLARIUS trial defined the prognostic “ATE score” comprising nine genes (unrelated to angiogenesis), which was not predictive for bevacizumab response [12]. Similar approaches are being pursued in the recurrent setting, where NF1 mutation was reported to be predictive for response to bevacizumab [17].

The present analysis is limited by its sample size, which might impede the detection of potentially small survival differences despite the liberal significance threshold of p = 0.1, and increase the susceptibility to confounding factors. Also, the GLARIUS trial cohort is restricted to *MGMT* promotor unmethylated glioblastoma while AVAglio included both *MGMT*-methylated and *MGMT*-unmethylated tumors [3]. However, the findings from Sandmann and colleagues were reported to be robust to adjustment for MGMT status in multivariable analysis [7]. Further, the frequency of patients in the standard arm receiving bevacizumab after first progression was considerably higher in the GLARIUS trial (GLARIUS: 66.7%, AVAglio: 31.1%), which might have contributed to the missing OS benefit in proneural tumors [3, 11]. Finally, the GLARIUS trial evaluated bevacizumab and irinotecan compared standard temozolomide, thereby deviating from AVAglio and RTOG 0825, where bevacizumab was combined with temozolomide [3, 4, 11].

Due to the controversial findings regarding the potential survival benefit of proneural glioblastoma from first-line bevacizumab and to settle this open question, we join the call for independent testing in the RTOG 0825 study [8–10].

## Data Availability

Restrictions apply to the availability of these data due to privacy restrictions.
